# The Impact of Recycled Neonatal Incubators in Nigeria: A 6-Year Follow-Up Study

**DOI:** 10.1155/2010/269293

**Published:** 2011-02-06

**Authors:** Hippolite Onyejiaka Amadi, Jonathan C. Azubuike, Uriah S. Etawo, Uduak R. Offiong, Chinyere Ezeaka, Eyinade Olateju, Gilbert N. Adimora, Akin Osibogun, Ngozi Ibeziako, Edna O. Iroha, Abdulhameed I. Dutse, Christian O. Chukwu, Eugene E. Okpere, Mohammed B. Kawuwa, Aliyu U. El-Nafaty, Sulyman A. Kuranga, Olugbenga Ayodeji Mokuolu

**Affiliations:** ^1^Bioengineering Department, Imperial College London, London SW7 2AZ, UK; ^2^Department of Paediatrics, University of Nigeria Teaching Hospital, Enugu 400001, Nigeria; ^3^Department of Paediatrics, University of PortHarcourt Teaching Hospital, PMB 6173, Port Harcourt 500102, Nigeria; ^4^Department of Paediatrics, University of Abuja Teaching Hospital, PMB 228 Abuja, Nigeria; ^5^Department of Paediatrics, Lagos University Teaching Hospital, PMB 12003, Lagos 101001, Nigeria; ^6^Department of Paediatrics, Aminu Kano Teaching Hospital, PMB 3452 Kano, Nigeria; ^7^Department of Paediatrics, Ebonyi State University Teaching Hospital, Abakaliki 480001, Nigeria; ^8^Department of Paediatrics, University of Benin Teaching Hospital, POB 1111, Benin-City 300001, Nigeria; ^9^Department of Paediatrics, Federal Medical Centre, Nguru 630001, Nigeria; ^10^Department of Paediatrics, Federal Medical Centre, PMB 0037 Gombe, Nigeria; ^11^Department of Paediatrics, University of Ilorin Teaching Hospital, Ilorin 240001, Nigeria

## Abstract

Nigeria has a record of high newborn mortality as an estimated 778 babies die daily, accounting for a ratio of 48 deaths per 1000 live births. The aim of this paper was to show how a deteriorating neonatal delivery system in Nigeria may have, in part, been improved by the application of a novel recycled incubator technique (RIT). Retrospective assessment of clinical, technical, and human factors in 15 Nigerian neonatal centres was carried out to investigate how the application of RIT impacted these factors. Pre-RIT and post-RIT neonatal mortalities were compared by studying case files. Effect on neonatal nursing was studied through questionnaires that were completed by 79 nurses from 9 centres across the country. Technical performance was assessed based on 10-indices scores from clinicians and nurses. The results showed an increase in neonatal survival, nursing enthusiasm, and practice confidence. Appropriately recycled incubators are good substitutes to the less affordable modern incubators in boosting neonatal practice outcome in low-income countries.

## 1. Introduction

Contemporary medical practice is anchored in advanced engineering technology mostly provided by the developed world. Most developing countries like Nigeria rely on over 90% of imported medical technology for the running of her healthcare delivery systems as has been noted in the literature [1]. This in itself poses a great limitation in the efficiency of the health systems in many developing countries because essential equipment are often insufficient, poorly managed, or difficult to replace. Thus in the face of a shaky world economy and lack of adequate funding for the continued importation of these technologies, the health care system is bound to fail with a consequent high mortality and morbidity [2, 3].

A typical Nigerian tertiary hospital would require a minimum of 20 functional incubators to adequately handle its high admission delivery resulting from high population of people within its catchment zones [4, 5]. Amadi et al. [6] reported that the neonatal centres of most of the Nigeria's premier tertiary hospitals were without an adequate number of functional incubators to handle their numerous referral cases. The hospitals were rather littered with carcases of old and obsolete models of incubators that had long been phased out by the manufacturers and hence without available spare parts for maintenance. The problem was not limited to obsolete incubators as the functionality of more current models was also compromised due to inefficient supply chain of spare parts. Many of these hospitals resorted to make-shift hot boxes and poorly serviced obsolete incubators that often led to uncontrollable heating, overheating some babies to death. The very old and crude method of “hot water bottles in cot” returned as a regular practice in place of functional incubators [5, 7, 8]. This led to diverse kinds of disasters, exposed nurses to different degrees of burns and some newborns died from spilling hot water [9, 10]. Thermal distress became a major player for high neonatal mortality exacerbated by a rate of 62% point-of-admission hypothermia as reported by Ogunlesi et al. [7]. Clinicians and nurses hence practiced with unprecedented frustration [5, 11, 12]. It has been shown that effective maintenance of thermoneutrality and appropriate humidification in neonates can enhance survival rates, while neonatal hypothermia is a major cause of rapid death in newborns [13–18]. However, it was impossible to equip the neonatal centres with adequate number of functional incubators due to poor funding and because the country depended on 100% importation of these at unaffordable costs. Services were epileptic in the few places where any of these incubators existed at all due to complete lack of system maintenance that could only be rendered by the manufacturers' foreign technicians. It became obvious that Nigeria needed some kind of locally manageable engineering approach to save the teaming population of her neonates for which an estimated 284,000 die each year or 778 per day [19]. To this end, Amadi et al. [6] developed the “recycled incubator technique”, reporting its cost-effectiveness and locally driven spare parts production. This paper presents how Recycled Incubators may have contributed to reverse a deteriorating neonatal delivery system in Nigeria.

## 2. Methods

### 2.1. Preintervention

**Figure 1:**
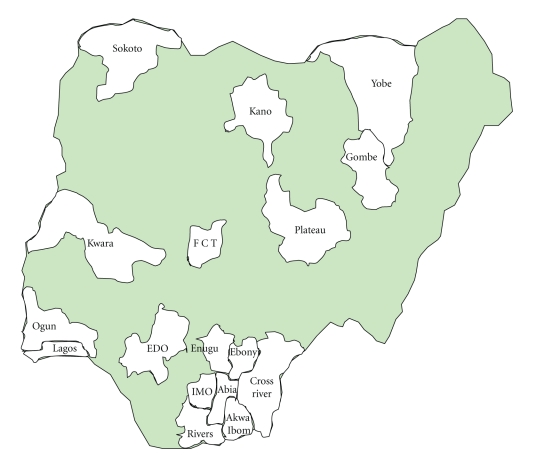
Distribution of the States of Nigeria that host Tertiary Hospitals where RIT intervention had been carried out. The more recent (less than 2 years) hospital entrants in the use of RIT were not included in this study because these lacked the necessary length of RIT service life required in the study.

This is a multicentre followup on a previous study by Amadi et al. [6] that developed the application of Recycled Incubator Technique (RIT) that was used to revamp various obsolete incubators in Nigerian tertiary hospitals. Fourteen of these hospitals, chosen on the basis of availability of a neonatal unit and the need to cover all the geographical regions of the country's tropical climate, were involved in this study, (see Figure 1). Prior to the introduction of the recycled incubators, various ideas as contributed from initial interviews and on-the-spot assessments were collated to generate effective solutions for the provision of adequate functional incubators at affordable costs to re-equip the hospitals. The systems so-recycled were used to completely re-equip the hospitals or supplement the relatively expensive modern incubators that were in short supply.

### 2.2. Recycled Incubator Technique

In RIT application, carcasses of dysfunctional incubators were reused by a careful reconditioning process. The canopies, access windows and doors, window blinds, filtration systems, and other peripheral assemblies were refashioned in simple designs that were locally producible. Temperature and humidity processing and air reconditioning assemblies, oxygen concentration assemblies, and safety devices were reorganised to fit the existing compartments of the particular model. These were achieved using open-market generic products. These were cost-competitively chosen and procured based on their output effects that were capable of substituting the requirement of various assembly components of the incubator model. Process reconfiguration, additional inter-connectivity, and assembling interfaces for all basic internal assemblies were designed, tailored to every specific model and adjusted to suit outputs from the procured components. Clinical trials and performance assessments of initial 11 units of the produced RIT incubators were rigorously conducted for six months at the Neonatal Units of the University of Nigeria, Enugu and Jos University Teaching Hospitals. This involved the first 1000 days of cumulative function [6]. Based on initial satisfactory performance of the RIT, it was introduced to other teaching hospitals to either re-equip or supplement the few modern ones in use. These include UITH Ilorin, LUTH Lagos, UPTH PortHarcourt, and FMC Owerri. Currently over 100 units of the systems are being used in more than 14 tertiary hospital centres, covering all geopolitical regions of Nigeria (Figure 1).

### 2.3. Six-Year Followup

The six-year followup performance assessment was conducted based on analyses of three indicators, (1) Impact of RIT systems on overall neonatal mortality, (2) RIT impact on nursing practice, (3) Technical evaluation of RIT functional parameters.

#### 2.3.1. Effect on Neonatal Mortality

The pre-RIT and post-RIT neonatal survivals and mortalities were directly assessed from the record books of 3 centres. This involved the assessment of at least 12 months of neonatal care before and after the introduction of RIT systems in each centre. This was rigorously done as each neonatal case was carefully examined to isolate admissions that involved hypothermic complications and required a possible intervention of an incubator at some stage. Most centres opted out of this retrospective study segment due to shortfalls in record book content such that cases of interest could not be effectively isolated.

Pre-RIT record examination extended only as far back as the number of available corresponding months of post-RIT records in the participated centres for a fair comparison. Figures noted in this exercise included (1) total overall number of babies admitted during the period, (2) total number of individual incubator-dependent neonates (IDNs) admitted, (3) total number of these IDNs that were successfully discharged, (4) total number of those discharged by patient carers' choice or referred to other centres, and (5) total number of deaths. These were applied to quantify the relative fraction the surviving neonates constituted based on the total number that was fully nursed (admission to discharge) at the centre. Poor performance of centres due to lack of functional equipment often leads to the failure of carers in choosing such centres for their patients. Therefore the relative overall number of babies admitted at the centres was applied as a contributing measure of carers' confidence. This application particularly ensured that increase or decrease in admission was not due to a growing/dwindling catchment population or change in payment structure that increased/decreased access to care during this period. Availability of functional incubators may also have a contributory role for a faster weight gain and hence earlier successful discharge. Therefore, the average lengths of stay before successful discharge were calculated and compared.

#### 2.3.2. Effect on Nursing Practice

Seventy-eight nurses in 9 hospital centres, mean (SD) of 8.7 (2.8), participated in a questionnaire-based assessment of the impact of RIT in neonatal nursing in their centres. Stringent measures were applied to ensure that only qualifying nurses from each centre assessed their practice experiences. Hospital nursing management in Nigeria often impose compulsory rotation of staff around the hospital wards with the exception of very few. Therefore to qualify as a questionnaire respondent, a nursing staff must have continuously worked in the neonatal centre for at least 12 months leading to RIT introduction and at least 12 months immediately following this. Four factors required were: to be assessed in the questionnaire according to the private opinion of each respondent. How has RIT assisted (1) your neonatal survival confidence, (2) actual neonatal survival, (3) your overall nursing enthusiasm, and (4) your desire to use more RIT incubators in your practice. They were asked to normalise their pre-RIT practice experience of these factors to 1 and hence score these against post-RIT experience in a scale of 0 to 10 (from very poor to very good); score “0” signifying “poorer post-RIT experience”, score “1” signifying “no perceived practice difference”, and “2–10” signifying “perceived increasing degree of better post-RIT experience”.

#### 2.3.3. RIT Technical Evaluation

This was conducted on 31 incubators that had completed a minimum of six years of continuous service in 6 tertiary hospitals across Nigeria. The method for this assessment followed a similar procedure as described in the literature [6]. Forty-two clinical attendants from the hospitals independently provided performance scores for RIT systems they use. These were on the following parameters: (a) set point accuracy, (b) maintainability (defined as ease of availability of system's spare parts and maintenance engineers in Nigeria), (c) ease of hood accessibility, (d) heating transient response, (e) sensitivity and response to erratic power supply, (f) humidification, (g) system aesthetic appreciation, (h) operation inherent noise, (i) response to high ambient temperatures, and (j) cost of system procurement. The scores were such that the ideal desirable level of an index was scored 10 and what was considered a very poor level was scored 0. Overall satisfaction of the performance of RIT systems was chosen between “very dissatisfied”, “dissatisfied”, “satisfied”, and “very satisfied”. There was no comparative performance assessment against the non-RIT incubators as most of the centres did not have functional non-RIT incubators with up to 6 years of continuous service. These were either reported to have been out of use for lack of spare parts or effective maintenance personnel. However, the relative presence of functional non-RIT incubators was noted in all centres and applied to compute the average RIT contribution towards the survival of Nigeria's incubator-dependent neonates.

## 3. Results

**Figure 2:**
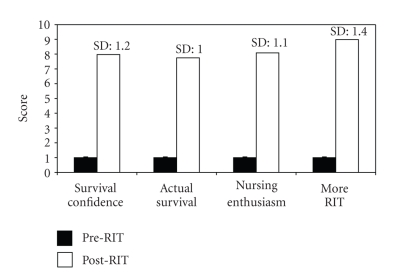
Impact of RIT on nursing practice experience from 9 hospitals centres; survey results with normalised prescores of 1 and postscores out of 0 to 10, *n* = 79 respondents.

**Figure 3:**
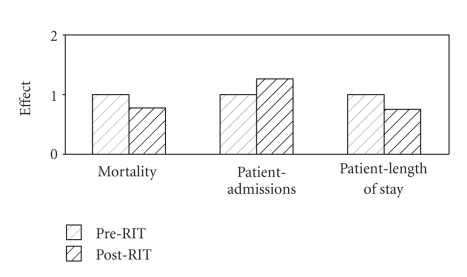
Average impact as extracted from hospitalisation case files.

Seventy-eight of an expected 86 questionnaires (91%) were fully completed and returned by the participating nurses. The returned questionnaires indicated that on average, nurses reported an overwhelming increase in their confidence and for their overall nursing enthusiasm with an average ranking of over 8 on the scale of 0 to 10 with 1 signifying “no perceived practice difference” (Figure 2). Actual retrospective measure of impact through patient files shows an average decrease in mortality by 22% (Figure 3) from 254/000 to 198/000.

**Figure 4:**
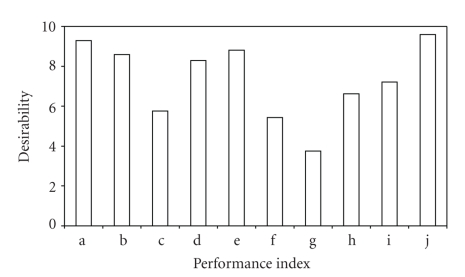
Mean scores of performance indices for RIT incubators (a) set point accuracy, (b) maintainability (defined as ease of availability of system's spare parts and maintenance engineers in Nigeria), (c) ease of hood accessibility, (d) heating transient response, (e) sensitivity and response to erratic power supply, (f) humidification, (g) system aesthetic appreciation, (h) operation inherent noise, (i) response to high ambient temperatures, and (j) cost of system procurement.

**Figure 5:**
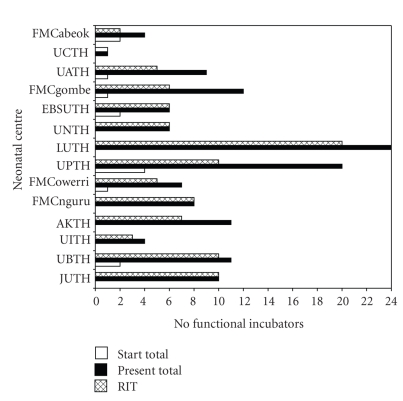
Total number of functional incubators at various centres. The three descriptive bars for each centre represent the number of functional incubators in each centre before RIT (total at start), the total number of current functional incubators (total presently), and finally, the number of those current functional incubators that are RIT; abbreviated centres are: University Teaching Hospitals at Jos (JUTH), Benin-City (UBTH), Ilorin (UITH), Kano (AKTH), Port Harcourt (UPTH), Lagos (LUTH), Enugu (UNTH), Abakaliki (EBSUTH), Abuja (UATH), Calabar (UCTH) and Federal Medical Centres at Owerri (FMCowerri), Nguru (FMCnguru), Abeokuta (FMCabeok), Gombe (FMCgombe).

All of the clinicians and nurses reported overall improvement in practice and better success rates in neonatal survival. Eight-eight percent of these believe that their perceived higher success rate was mainly due to the incubation boost from RIT systems as most of the other clinical factors did not change significantly. Four of the providers (9.1%) however think that other factors have equal contribution to the improved success rates, including recently introduced use of apnoea monitors in their respective centres.  Figure 4 shows a summary of the average scores for the various indices of RIT system assessment by the users. On overall performance satisfaction, 37 out of 42 (88.1%) of users were “very satisfied” and the rest were “satisfied”. There was no reported dissatisfaction.  Figure 5 shows three informative bars describing availability of functional incubators in each centre; these are (1) the total number of functional incubators at the introduction of RIT systems (total at start), (2) the total number of functional incubators current at the time of this study (total presently), and finally (3) the total number of those current incubators that are powered by RIT. At the present, only the University of PortHarcourt (UPTH) and Lagos University (LUTH) Teaching Hospitals have at least 15 units of functional systems each. For the 14 hospitals listed, there are a total of 133 functional incubators. This includes 99 RIT incubators and 34 others. None of the initial non-RIT incubators that were met in functional condition at the introduction of RIT systems in the 6 centres was found functional during this study.

## 4. Discussion

The World Health Organisation (WHO) states that only six countries, including Nigeria, jointly contribute 50% of annual global deaths of children of less than five years of age [20]. This figure is largely a reflection of the terrifically high neonatal mortality within the country [19]. Our collaborative experience shows that some of the neonatal deaths in the referral hospitals occur as a result of inefficient maintenance of neonatal thermo-neutrality as effective practice would require. This study agrees with others and shows that the blame is partly due to lack of adequate supply of functional incubators and other essential equipment needed in the hospitals [5, 21, 22]. It is arguably correct that facility deliveries and kangaroo warming practices (direct skin to skin contact with the mother) during transport are factors that could help decrease the high point-of-admission hypothermia. However, cultural and demographic situations in Nigeria, for example, make these factors only partially effective as poverty, long travel distances and ineffective motorized transport still work together to cause many of the babies to arrive at facilities weak or hypothermic. The prevalence of these societal factors means that availability of an adequate number of functional incubators cannot be compromised.

We set out to apply the idea of Recycled Incubator Technique (RIT) to supplement the existing incubators in the neonatal centres of fourteen participating tertiary hospitals in Nigeria. The generic-components approach adopted in RIT incubators allowed the technique its robust ability to easily accept a switchover at any time to another generic, more readily available and cost-effective unit of a component. This also enables RIT incubators to remain operational irrespective of an economic downturn affecting the producers of any of its functional components. Its operational design, tailored to suit the common traditional constraints of environmental, power, and social factors of low-income countries makes this a user-friendly piece of equipment.

Jos University Teaching Hospital (JUTH) applied the initial 10 systems in 2003, and these have remained the only functional systems in use in the hospital until this current study. The 6-year followup assessment of system performance in all the participating hospitals shows that the system is reliable with a high user-satisfaction rating. This may have, in no small measure, contributed to the reversal of a once dwindling practice enthusiasm and high neonatal mortality in these hospitals. The hospitals listed in this study constitute most of the premier Nigerian hospitals and hence reflect the national situation. This suggests that RIT might have provided an unprecedented contribution towards the saving of neonates in Nigeria. Its usage is also well represented across the country and constitutes over 74% of all the functional incubators in the study hospitals, hence significantly contributing to the resolution of a seemingly national problem. Evidently, from the report of Ademola Oyedeji et al. in 1983 to that of Ogunlesi et al. in 2008, Nigerian hospitals have many incubator carcasses but fewer than three functional units for application at one time [5, 6, 12]. RIT has however changed this trend in the participating hospitals with many of these consistently operating with over 10 functional incubators. These include the Lagos University Teaching Hospital that currently operates with up to 24 functional incubators.

Direct measurements of neonatal mortality (Figure 3) appear to, in part, agree with the perception of the nurses (Figure 2). However, it is understood that careful parametric isolation needed to have been initially defined and prospectively measured during intervention to be able to ascertain the exact incubator contributions towards individual survival. We therefore can only report that these findings correlate with and contributed to a positive clinical impact of the RIT systems. Furthermore, functional incubators may have also helped to enhance carers' confidence and motivated increased care-seeking behaviour of patients as evidenced by the increased number of admissions.

## 5. Conclusions

In the present 6-year followup study, RIT systems were found to comprise three-quarters of functional incubators in 14 Nigerian centres. System performance was rated high; provider satisfaction and confidence were much improved; neonatal mortality coincidentally declined. RIT may be a way forward for the rest of the public hospitals in low-income countries especially as high neonatal mortality in these may, in part, be due to the short supply of functional incubators. The success of RIT demonstrates that locally manageable and appropriate technological solutions could apply to improve Nigeria's rating in infant and neonatal mortality. RIT could be a sustainable application in low-income countries if used incubators from developed countries were donated for recycling. This study may also act as a challenge and motivation for engineers from low income countries to get involved in supplementing imported technologies to make the healthcare delivery system of their countries more functional and affordable.

##  Conflict of Interests

The authors declare that they have no competing interests.

##  Authors' Contributions

H. O. Amadi conceptualised and designed the RIT application; he also coordinated and contributed at all levels of the present work, from study design to article submission. H. O. Amadi, J. C. Azubuike, U. S. Etawo, U. R. Offiong, C. Ezeaka, O. Eyinade, G. N. Adimora, A. Osibogun, N. Ibeziako, E. O. Iroha, A. I. Dutse, C. O. Chukwu, E. E. Okpere, M. B. Kawuwa, A. U. El-Nafaty, S. A. Kuranga, and O. A. Mokuolu all contributed equally to this work on initial installations in their respective hospitals, system monitoring, system optimization, study reviews and manuscript readiness.
